# Relaciones y procesos de familias en confinamiento por COVID-19 narradas por madres*

**DOI:** 10.15649/cuidarte.2347

**Published:** 2023-05-27

**Authors:** Ana Luisa Pedraza-Valdez, Karla Aimee Maldonado-Cervantes, Beatriz García-Solano

**Affiliations:** 1 . Instituto Mexicano del Seguro Social. Puebla, México. Email: anilupv_911@gmail.com Instituto Mexicano del Seguro Social Instituto Mexicano del Seguro Social Puebla Mexico anilupv_911@gmail.com; 2 . Benemérita Universidad Autónoma de Puebla. Puebla, México. Email: aimee150294@hotmail.com Benemérita Universidad Autónoma de Puebla Benemérita Universidad Autónoma de Puebla Puebla Mexico aimee150294@hotmail.com; 3 . Benemérita Universidad Autónoma de Puebla. Puebla, México. Email: bgsolano@hotmail.com Benemérita Universidad Autónoma de Puebla Benemérita Universidad Autónoma de Puebla Puebla Mexico bgsolano@hotmail.com

**Keywords:** COVID-19, Relaciones Familiares, Teoría Fundamentada, Adaptación Psicológica, Salud de la Familia, Transición, COVID-19, Family Relations, Grounded Theory, Adaptation, Psychological, Family Health, Transition, COVID-19, Relações Familiares, Teoria Fundamentada, Adaptação Psicológica, Saúde da Família, Transição

## Abstract

**Introducción:**

El estado de alarma que provoco COVID-19, obligó a que se tomaran medidas sociales de restricción, esperando reducir los contagios, incluyendo el confinamiento, que impactó diversos aspectos de la vida humana, ha tenido efecto considerable en la dinámica familiar, provocando que permanezcan en casa acrecentando el afrontamiento de las situaciones de crisis, experimentando un proceso de transición a nuevas formas de vida. Objetivo: analizar las relaciones y procesos que viven las familias ante el confinamiento por COVID-19 narradas por las madres.

**Materiales y métodos:**

estudio cualitativo de teoría fundamentada, se realizaron entrevistas a profundidad a cinco madres de familia.

**Resultados:**

los resultados obtenidos emergieron de una categoría central “adquisición de nuevas formas de vida” y tres categorías que la sustentan: 1. Afrontando el inicio de la pandemia; 2. Adaptando la nueva normalidad en familia y 3. Adoptando las consecuencias de la pandemia en la vida cotidiana.

**Discusión:**

El proceso que han mostrado las familias mexicanas ante el COVID-19 se vive diferente al de otros países, la convivencia en los hogares fue de las afectadas ya que se compromete la libertad con la que viven día a día.

**Conclusión:**

Las participantes expresaron que durante la pandemia por COVID-19, la familia vivió un proceso de transición para poder adquirir nuevas formas de vida, las cuales incluían hábitos, costumbres y estructuras que cubrieran sus necesidades requeridas en ese momento, este proceso incluyó diferentes etapas (afrontamiento, adaptación y adopción).

## Abstract (en)

**Introduction:**

The state of emergency caused by COVID-19 forced the adoption of social restriction measures to reduce contagion, including lockdowns. They impacted various aspects of human life and considerably affected family dynamics by causing families to remain at home, increasing the coping of crisis, and experiencing a transition to new ways of living.

**Objective:**

To analyze the relationships and processes experienced by families during the COVID-19 lockdown as told by mothers.

**Materials and Methods:**

A qualitative grounded theory study was conducted using in- depth interviews with five mothers.

**Results:**

The results obtained emerged from a core category, “acquisition of new ways of living,” and three categories that support it: 1) Coping with the pandemic onset, 2) adapting to the new normal in the family, 3) embracing the pandemic consequences in everyday life.

**Discussion:**

The process that Mexican families have gone through with COVID-19 has been different from that of other countries; living together at home was affected since their freedom to live day to day as they did was compromised.

**Conclusion:**

The participants expressed that, during the COVID-19 pandemic, the family underwent a transition process in order to acquire new ways of living, which included habits, customs, and structures that met their.

## Resumo

**Introdução:**

O estado de alarme causado pela COVID-19 obrigou à adoção de medidas de restrição social, na esperança de reduzir o contágio, incluindo o confinamento, que impactou vários aspetos da vida humana, tem tido um efeito considerável na dinâmica familiar, fazendo com que a permanência em casa aumente o enfrentamento com situações de crise, vivenciando um processo de transição para novos modos de vida. Objetivo: analisar as relações e processos que as famílias vivenciam diante do confinamento por COVID-19 narrado pelas mães.

**Materiais e métodos:**

estudo qualitativo de grounded theory, foram realizadas entrevistas em profundidade com cinco mães. Resultados: os resultados obtidos emergiram de uma categoria central “aquisição de novas formas de vida” e três categorias que a sustentam: 1. Enfrentando o início da pandemia; 2. Adaptando-se ao novo normal em família e 3. Abraçando as consequências da pandemia na vida cotidiana.

**Discussão:**

O processo que as famílias mexicanas mostraram diante do COVID-19 é vivido de forma diferente do de outros países, a convivência em casas foi uma das afetadas, pois a liberdade com a qual convivem no dia a dia está comprometida.

**Conclusão:**

Os participantes expressaram que durante a pandemia do COVID-19, a família passou por um processo de transição para poder adquirir novos modos de vida, que incluiu hábitos, costumes e estruturas que cobriam suas necessidades exigidas naquele momento, esse processo incluiu diferentes etapas (enfrentamento, adaptação e adoção).

## Introducción

El virus del SARS-CoV2 surgió en Wuhan, China a finales del año 2019, se extendió por todo el mundo, el 11 de marzo de 2020 COVID-19 fue declarada por la OMS como pandemia. En México el primer caso se reportó el 27 de febrero de 2020^1^. De acuerdo a datos epidemiológicos a nivel mundial existen 550,815,446 casos positivos y ha cobrado la vida de 6,341,424 personas (corte al cierre 05 julio 2022). En México existen 6,093,835 casos positivos y la cifra de defunciones por esta enfermedad es de 325,793 muertes. (corte al cierre 05 julio 2022)^2^. En Puebla el primer caso se registró el 10 de marzo de 2020, y ha cobrado la vida de 140,921 personas (corte al cierre 2 febrero 2022)^3^.

El estado de alarma que provoco esta enfermedad obligó a que se tomaran medidas sociales de restricción, como el uso de cubrebocas, lavado de manos con más frecuencia, uso de gel antibacterial, mantener sana distancia (1.5 metros); para evitar aglomeraciones esperando reducir los contagios, se aplicó el confinamiento domiciliario, y la mayoría de las actividades se comenzaron a realizar desde el hogar^4^.

El confinamiento ha impactado diversos aspectos de la vida humana, el tomar clases a través de una computadora, una tablet o teléfono celular, actividades que no son esenciales, pero si necesarias se están realizando desde casa “home office”, medidas que tienen como fin, evitar contagios, así como mantener la salud de los estudiantes y trabajadores evitando el contagio secundario entre contactos cercanos y/o familiares, la pandemia no solo ha puesto en riesgo el estado de salud físico, también existe un impacto económico y social, además de que el confinamiento ha tenido efecto considerable en la dinámica familiar ya que ha provocado que permanezcan en familia realizando sus actividades cotidianas^5^^,^^6^.

De acuerdo a la Organización de las Naciones Unidas [ONU] 2019^7^, no se cuenta con un número de familias existentes a nivel mundial, sin embargo, si se toma el hogar como unidad de análisis, poco más de un tercio de los hogares del planeta (38 %) están formados por una pareja con hijas e hijos de cualquier edad. En el caso de México de acuerdo con la Encuesta Nacional de Ingresos y Gastos de los Hogares 2018 (ENIGH)^8^ en México hay 34,744,818 hogares, de estos, 28.7 % encabezados por mujeres y 71.3 % por hombres. El tamaño promedio del hogar es de 3.6 personas, en estos la edad promedio de la jefa o jefe alcanza los 49.8 años. Los hogares familiares comprenden el 88.1 % del total de hogares del país^9^.

El confinamiento en familia ha provocado el afrontamiento de situaciones de crisis, para poder comprender el proceso de transición que viven las familias durante la pandemia se realiza la presente investigación basada en el interaccionismo simbólico, propuesto por Blumer (1986)^10^ que propone lograr la comprensión del significado de los fenómenos sociales, concibe a la sociedad como la interacción simbólica entre individuos y al ser humano como un constructor de significados en torno a los procesos de interacción.

En la pandemia, la familia es el primer núcleo afectado y quienes están afrontando la difícil situación, se desea generar nuevo conocimiento e incitar a los investigadores a realizar estudios sobre familia ante situaciones que impliquen un cambio en la dinámica y rutinas familiares^11^. Debido a esta situación surge la pregunta de investigación ¿Cómo es la dinámica familiar que se vive ante el confinamiento por COVID-19 desde la perspectiva de las madres? Con el objetivo de analizar las relaciones y procesos que viven las familias ante el confinamiento por COVID-19 narradas por las madres.

## Materiales y Métodos

Estudio cualitativo que busca analizar las relaciones y procesos que viven las familias ante el confinamiento por COVID-19 narrado por las madres, las entrevistas se realizaron a las madres ya que las mujeres toman decisiones y dirigen la toma de decisiones en sus familias, además, en su mayoría les corresponde el trabajo doméstico y cuidados no remunerados^7^. Como base metodológica se utilizó teoría fundamentada que proveniente del interaccionismo simbólico utilizado para derivar sistemáticamente teorías sobre el comportamiento humano y el mundo social, con una base empírica^12^. La Teoría Fundamentada ofrece una manera de representar la realidad que arroje luz o un entendimiento sobre lo estudiado. Por tanto, a través de los procedimientos analíticos, se genera una teoría derivada de datos recopilados de manera sistemática y analizados mediante la generación de principios y procesos heurísticos basados en el descubrimiento de conceptos y categorías de los significados expuestos, que puedan dar como consecuencia una teoría emergente de los hallazgos analizados^13^.

Se realizó un primer contacto con las participantes a través de la difusión de invitaciones a charlar sobre la experiencia ante el confinamiento por medio de redes sociales, a los interesados se les proporcionó la información y se programaron citas para realizar videollamadas por plataformas digitales. Se entrevistó a cinco mujeres que buscan representar de la mejor manera a la población, todas con diferentes ocupaciones, estado civil, que fueran madres, se encontraran entre la edad de 25 y 59 años, que sus hijos fueran menores de 12 años, que trabajaran desde casa o no trabajaran. Se programó en primera instancia una entrevista exploratoria, donde se comentó sobre la aceptación de participar en el proyecto, por medio del consentimiento informado, se hizo énfasis en la confidencialidad de los datos proporcionados y de utilizar un nombre ficticio para proteger su identidad, se obtuvieron datos socioculturales y se formó un vínculo para generar confianza con el participante.

Las investigadoras fueron enfermeras graduadas quiénes fungieron como entrevistadoras quienes realizaron cinco entrevistas formales con un solo encuentro, se formularon dos preguntas detonadoras ¿cuál ha sido su experiencia en este confinamiento? y ¿Cómo es la dinámica de su familiar a raíz de esta pandemia? Las entrevistas tuvieron una duración total de 215 minutos; se llevaron a cabo en los domicilios de los participantes vía electrónica, mediante la plataforma Google meet. Para el registro fiel de las entrevistas los participantes permitieron la grabación de las videollamadas donde se utilizaron dos laptops marca Hp modelo ENVY m4, además se hizo uso de memos, en donde se anotaron palabras claves para poder retomarlas en las entrevistas y no interrumpir a las participantes, la información de los memos fue triangulada entre las tres investigadoras. Cada una de las entrevistas fueron trascritas en el programa Microsoft Office Word 2016, obteniendo un total de 45 cuartillas y 12,193 caracteres. Con la información recabada se obtuvo la saturación de las categorías; mediante el uso del software de Microsoft Excel se realizó el análisis de los códigos iniciales respecto a su frecuencia lo que permitió observar sus combinaciones co-textuales para destacar la significancia de cada código focal generado.

**Figura 1:**
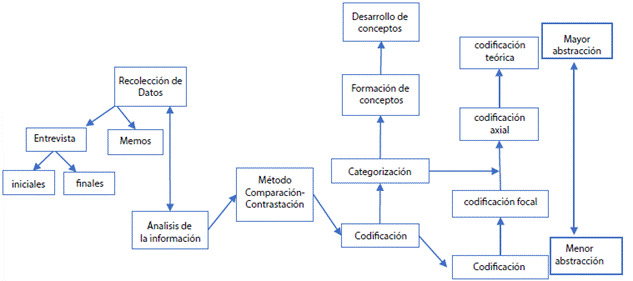
Esquematización de Recolección y Análisis de los datos

El análisis de los datos se llevó a cabo con lo propuesto por Strauss y Corbin^14^^,^^15^ el cual consistió en: 1. Nivel Textual, a través de documentos escritos, se asignaron códigos, definiciones y memos (codificación abierta); 2. Nivel conceptual, se agruparon códigos y memos con características comunes, de acuerdo a las relaciones entre ellos y al nivel de generalización (codificación axial y focal); y 3. Nivel teórico sustantivo, se realizó un análisis posterior para observar la repetición de códigos, de las cuales se obtuvieron nuevas categorías cada vez más abstractas conectadas a los códigos y la categoría central (codificación axial y focal) (Figura 1).

Durante el análisis de datos se consideraron los criterios de calidad propuestos por Lincoln y Guba (1985)^16^, que consiste en: 1) credibilidad, se realizó la trascripción literal de la entrevista semiestructurada, de una forma detallada de la voz de los participantes, sistema de codificación para representar expresiones faciales y silencios; 2) transferibilidad, el fenómeno de estudio ha sido bien descrito para que los lectores puedan evaluar la aplicabilidad de los datos a otros conceptos, se describe el fenómeno de estudio en tiempo, lugar y persona; 3) consistencia, se describe el proceso riguroso del análisis de datos y la recolección de la información; 4) confirmabilidad, los datos representan la información que los participantes proporcionaron y la interpretación de estos no fueron interferidos por el investigador. La recolecta y análisis inicial de los datos se realizó por dos investigadores, los códigos teóricos fueron validados por siete investigadores y los participantes validaron finalmente las definiciones y el proceso propuesto.

El presente estudio se apegó a las disposiciones del Reglamento de la Ley General de Salud en Materia de Investigación (SS, 2021)^17^ del Título Quinto, Investigación para la Salud, Capitulo Único, Articulo 100, fracción IV la investigación se ajustó a los principios científicos y éticos, se contó con el consentimiento informado de los participantes en quienes se realizó la investigación. Se tomaron en cuenta los principios éticos de enfermería como beneficencia y no maleficencia, fidelidad; se creó confianza y se mantuvo la confiabilidad entre los investigadores y los participantes, veracidad; los investigadores tienen el principio ético de decir la verdad, no mentir a los participantes de este estudio, confidencialidad; este principio ético salvaguarda la información de carácter personal que se obtuvo de las entrevistas a los participantes y se mantendrá de manera confidencial, bajo resguardo de los investigadores.

## Resultados

Participaron cinco madres cuya edad osciló entre los 27 y 41 años, los integrantes de su familia fueron de 3 a 6, con hijos preescolares o escolares, todas en situación laboral de desempleo por pandemia y home office; casadas, viudas o madres solteras, los roles que desempeñan son de madre, abuela y proveedora (Tabla 1).

**Tabla 1:** Características de los informantes

Núm. de entrevista	ID de la madre	Edad	Núm. Integrantes	Núm. Hijos	Situación	Estado Civil	Rol
1	PV	27	4	2	Desempleada por pandemia	Casada	Madre
2	PL	41	5	1	Home Office	Viuda por pandemia	Madre/ abuela/ proveedora
3	PA	31	4	2	Desempleada por pandemia	Casada	Madre
4	PC	33	3	1	Desempleada por pandemia	Casada	Madre
5	PVB	30	6	2	Home Office	Madre soltera	Madre/hija/ proveedora

Los resultados obtenidos de las dos fases de análisis se presentan a continuación. En la codificación inicial surgieron 88 códigos iniciales de los cuales se clasificaron los más frecuentes y significativos obteniendo cinco códigos focales (adaptando, enfrentando, enfrentando, adoptando y añorando)^15^; en la codificación teórica y después de emplear el Método de Comparación Constante (MCC) surgen tres códigos axiales (enfrentando el inicio de la pandemia, adaptando a la nueva normalidad en familia y adoptando las consecuencias de la pandemia en la vida cotidiana) y tres códigos teóricos (afrontando el inicio de la pandemia, adaptando a la nueva normalidad en familia y adoptando las consecuencias de la pandemia en la vida cotidiana) que dan origen a una categoría denominada “Adquisición de nuevas formas de vida” (Tabla 2).

**Tabla 2:** Método de Comparación Constante

Método de Comparación Constante (MCC)			Codificación Teórica		
Condiciones	Acción o interacción	Consecuencia	Código Axial	Código Teórico	Categoría
Expresan la manera en que impactó el inicio de la pandemia en las diferentes esferas de su vida	Existen cambios en los integrantes de la familia que modifican sus sentimientos, dinámica familiar, pensamientos, al vivir el inicio de la pandemia	Quejando Apoyando Preocupando Temiendo Responsabilizando Desanimando Angustiando Asimilando Convenciendo Esperanzando Estresando Desmotivando Negando	Enfrentando el inicio de la pandemia	Afrontando el inicio de la pandemia	Adquisición de nuevas formas de vida
Mencionan el proceso de ajuste vivido ante el confinamiento en familia	Existen cambios en el rol, la rutina, las actividades, el trabajo, así como en las medidas de higiene	Estrés Frustración Responsabilidad Aprovechamiento	Adaptando a la nueva normalidad en familia	Adaptando a la nueva normalidad en familia	Adquisición de nuevas formas de vida
Manifiestan las repercusiones existentes en su vida en familia después de vivir el inicio de la pandemia	Se reconoce la manera en que están haciendo frente a la pandemia	Extrañando Agradeciendo Quejando Desesperando Añorando Conformando Solucionando Aceptando Aliviando Concientizando Asimilando Empatizando	Adoptando las consecuencias de la pandemia en la vida cotidiana	Adoptando las consecuencias de la pandemia en la vida cotidiana	Adquisición de nuevas formas de vida

**Figura 2:**
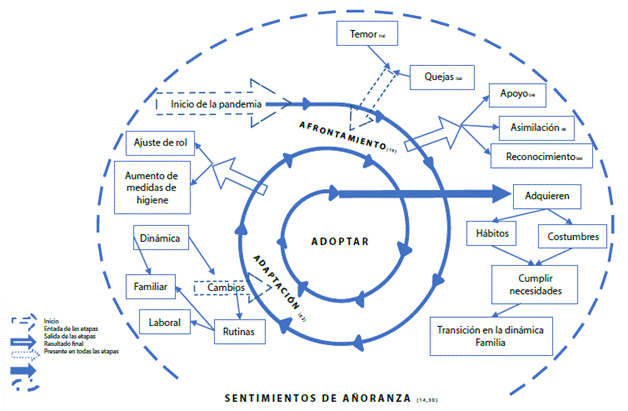
Proceso de transición de la familia ante la pandemia por COVID-19. Nota: (n...)= Número de veces que se repitieron los códigos iniciales durante las entrevistas.

Se obtuvo una categoría central “adquisición de nuevas formas de vida” de la cual se desprendieron tres subcategorías, que originan y contribuyen a la categoría central: 1. “afrontando el inicio de la pandemia” 2. “adaptando a la nueva normalidad en familia” y 3. “adoptando las consecuencias de la pandemia en la vida cotidiana” (Figura 2). Durante el suceso caótico provocado por el COVID-19 las familias viven diversos procesos, uno muy importante al que se someten, es el de la transición, entendido como un proceso de transformación, que tiene una secuencia y está integrado por etapas que dan como resultado la adquisición de nuevos hábitos, costumbres y estructuras familiares.

En la etapa inicial del proceso de transición se observa un impacto en la salud mental de las personas en el que destacan sentimientos negativos como el temor, quejas, miedo e incertidumbre provocando que las personas afronten esta situación mediante sentimientos positivos como el apoyo entre los integrantes de la familia, la asimilación de la situación y el reconocimiento del porque es importante realizar cambios en la dinámica y en las rutinas familiares, así como laborales para poder hacer frente a la pandemia, estos cambios pueden llegar a generar actitudes negativas como estrés y frustración lo que da paso a la segunda etapa denominada adaptación donde se busca un ajuste familiar haciendo cambios en el comportamiento, los roles y rutinas, alcanzando la tercera etapa de la transición en la familia denominada adopción, en esta etapa la familia ha pasado por repercusiones en su vida diaria, lo que ha permitido adquirir (apropiarse o aprehender) de hábitos, costumbres y estructuras que resuelven las necesidades de la vida cotidiana y ayuda a poder cumplir las necesidades de la familia ante la nueva normalidad.

Es durante este proceso donde la familia está en constante cambio y desconocen lo que va a suceder después, en las diversas etapas experimentan sentimientos de añoranza a la cotidianeidad de las actividades realizadas por la familia y sus integrantes antes de la pandemia, haciendo alusión que en algunas ocasiones les gustaría regresar a cómo era la vida antes. El proceso expresa no solo las consecuencias negativas de la pandemia, si no también evidencia las emociones y actitudes en lo positivo, donde se permite observar los cambios que se vinculan a la estructura de la familia.

### Adquisición de nuevas formas de vida

Proceso por el cual la familia adquiere nuevas formas de vida, hábitos, costumbres y roles mismos que surgieron de imprevisto, ya que al vivir con incertidumbre el inicio de la pandemia, llegaron a descontrolar toda su rutina, y tuvieron que improvisar la manera de cubrir sus necesidades consecuentes, buscando hacerlo de manera consciente.

### Afrontando el inicio de la pandemia

Es la manera en que hacen frente a la situación que impactó en la vida de la familia, modificando su dinámica, experiencias vividas, pensamientos, reacciones y sentimientos negativos como miedo, angustia, estrés negación y desamino. Las familias también demuestran apoyo y responsabilidad en los actos y actividades que realizan, teniendo sentimientos de esperanza a pesar de lo vivido durante la pandemia. Los elementos que la componen son los sentimientos negativos, como: miedo, angustia, estrés, negación y desánimo; también sentimientos positivos que pueden ser: apoyo, responsabilidad y conciencia.

En este estudio realizado en madres de familia mencionan que en la etapa de afrontamiento al inicio de la pandemia les generó en primera instancia sentimientos negativos que provocaron que la dinámica familiar se viera afectada, mostraron negación pero conforme pasaban los días enfatizaron en que tenían miedo ya que desconocían lo que iba a pasar al siguiente día; también comenzaron a concientizar que las medidas de higiene, el distanciamiento social y el aislamiento eran necesarias para sobrellevar la pandemia y así comenzaron a ser más responsables y a apoyarse entre ellos. Aunado a esto, también se identificaron sentimientos de añoranza, cuando tenían la oportunidad de mostrarlos lo hacían y deseaban volver a la vida de antes.

*PA: “Miedo a contraer la enfermedad y pues miedo a que te pase algo. El hecho de decir “si me pasa algo ¿quién va a cuidar a mis niños?” o si ellas se enferman… ese miedo más que nada. Igual a mi esposo que se va al trabajo, también es el miedo de: “¿y si se contagia?” Más que nada el miedo a que nos suceda algo” (entrevista tres, página 3, línea 9 a 12)*

*PC: “Sí, pues por video llamada. Y pues sí vamos, por lo menos cada mes. Y ya pues siempre que vamos nos reunimos con mis hermanos, siempre, siempre hay reunión. Mal hecho, ¿verdad? Pero sí” (entrevista cuatro, página 8, líneas 7 y 8)*

*PL: “-Fue muy difícil, tuve meses con esa depresión, con esa ansiedad y pues más o menos la he ido superando, pero ha sido muy difícil para mí. Ya hicimos el año del confinamiento y para mí va a ser, pues, doloroso, porque yo perdí a mi esposo por COVID. -…me ha apoyado mi familia, mi hija. Ha estado al pendiente de mí, de mis depresiones, de repente de mis crisis. Este, también voy a otras terapias con otra psicóloga y pues también, mis hermanas también me han apoyado mucho. Mis compañeros del trabajo, también han estado al pendiente de mí y de mi salud física y psicológica.” (entrevista uno, página 1, línea de 13 a 16 y página 4, línea 7 a la 10)*

*PC: “Pues sí, a veces es un poco frustrante porque, pues soy mamá y, o sea, siempre me he dedicado a negocios propios, a veces mi… mm, como se dice, como mi aliviane, a veces es salir un poco a, un fin de semana o ir a comer y que ahorita ya no pueda a veces sí es un poco complicado. Sí creo que es de lo que siento que, como que me frustra un poco.” (entrevista cuatro, página 2, línea 17 y página 3, línea 1 a 3)*

*PL: “Nunca me voy a acostumbrar a estar aquí en casa, a trabajar desde casa. Yo creo que no, nunca me voy a acostumbrar” (entrevista uno, página 2, línea 4 y 5)*

### Adaptando la nueva normalidad en familia

Es la búsqueda del ajuste familiar ante el confinamiento por covid-19, pasando por cambios en el comportamiento, rol y rutinas; las familias desarrollan estrategias que les permiten enfrentar las situaciones estresantes, lo que a su vez genera la adopción de hábitos, costumbres y roles. Los elementos que la integran son cambios en el rol, rutinas y trabajo, algunas actitudes positivas como: responsabilidad, tolerancia y aprovechamiento, actitudes negativas como: estrés, frustración y resignación.

Durante el análisis de los datos se pudieron identificar que las familias, cambiaron los roles, dejaron en el olvido algunas costumbres para poder llevar a cabo las restricciones que impusieron organismos internacionales y nacionales, así como el gobierno: federal, estatal y municipal; algunas medidas repercutieron en sus actividades laborales, sociales y escolares, mismas que generaron que se presentara estrés, frustración, así como resignación ante los cambios que tuvieron que realizar debido a la pandemia, sin embargo, conforme avanzaban los días, también se generaron situaciones positivas como el aprovechamiento de la situación, la unión y convivencia familiar así como la tolerancia entre los integrantes de la familia, pero siempre mostrando sentimientos de añoranza.

*PVB: “Pues debido a la situación económica, tuve que pasarme a la casa de mis papás, porque yo estaba rentando, entonces a mí me redujeron el salario al 50% y tuve que, este, mudarme a la casa de mis papás”*

*PL: “…cambiaron los roles. Mi hija se dedicaba a sus hijas. En esta pandemia ella se dedicó a trabajar y yo me dediqué a apoyarla, a cuidar a mis nietas y a las labores de casa. Sí, cambiaron los roles” (entrevista uno, página 5, líneas 6 a 8)*

*PV: “…antes dejaban dos tareas por semana y ahora dejan siete tareas por semana; y a veces tanto yo como ella nos desesperamos. Y ahora cómo me quede yo sin empleo… Pues…si me desesperaba al inicio, bueno de todos modos ahorita de me siento desesperada la verdad. (entrevista uno, página 4, líneas 15 a 17)*

*PA: “Y ahora la dinámica es diferente. Llega mi esposo con las compras porque él va solo y llega y nos ponemos a desinfectar todo, bueno dormimos primero a las niñas si no nos dejan, entonces por lo regular va al super en la tarde noche, regresa y pues entre los dos nos ponemos a lavar y guardar las cosas. Y…antes no había esa dinámica” (entrevista tres, página 5, líneas 14 a 17)*

*PV: “De me siento desesperada la verdad. Pero pues es de que se despierten de que bajemos a desayunar, de que jueguen. Antes decía yo “pobrecitos, se aburren, les voy a buscar actividades”, pero ahorita… ya, ya que jueguen, que tiren, que hagan lo que quieran, porque es desesperante estar así” (entrevista uno, página 4, líneas 1 a 4)*

*PC: “…yo lo veo a él un poco como león enjaulado por estar encerrado. Ya quiere, él ya quiere como a regresar a oficina también”*

*PA: “Él se quedaba aquí en el home office y a él le daba mucho sueño así de que, o sea no es lo mismo irse a la oficina, estar ahí interactuando a estar aquí en casa y escuchar que la niña está gritando y pues fue difícil” (entrevista tres, página 5, líneas 2 a 4)*

*PL: “Para fortuna de mi hija, ella emprendió un negocio y yo creo que a ella sí le preguntamos cómo ve la pandemia, ella dirá que estuvo bien, porque afortunadamente a ella le fue muy bien en su negocio” (entrevista uno, página 5, líneas 14 a 16)*

*PV: “Yo tampoco soy de las que dicen “ay, pues los voy a sacar al parque”, a mí no me gusta pues arriesgar a mi familia, por eso mismo nos mantenemos alineados a lo de quedarnos confinados y salir solamente cuando sea necesario” (entrevista uno, página 2, líneas 2 a 4)*

*PVB: “Y sí llegábamos a salir, al llegar a casa sanitizarse, eh, el tapete, este bueno el sanitizarse los zapatos. Inmediatamente alguien que llegue, el baño se encuentra a lado de la puerta, entonces el lavado de manos, este, sí vamos al super pues empezamos a sanitizar todo, este… el carro, eh, pues sí, sí optamos por todos estos protocolos que marcan las instituciones, entonces pues técnicamente creo que sí llevamos a cabo todas estas medidas.” (entrevista cinco, página 7, líneas 7 a 11)*

### Adoptando las consecuencias de la pandemia en la vida cotidiana

Se define como la manera en la que las personas se apropian de actividades que resuelven las necesidades de la vida cotidiana adquiriendo hábitos, costumbres y estructuras, se apropian de los cambios adquiridos como consecuencia de la pandemia. Los elementos que la integran pueden ser manifestaciones positivas como: solucionando, aceptando, aliviando, concientizando, asimilando, empatizando, agradeciendo y valorando; pero también puede haber manifestaciones negativas como: quejas y desesperación.

Al analizar el proceso de cada una de las etapas, se logró identificar que en la tercera etapa denominada adoptando las consecuencias de la pandemia en la vida cotidiana las madres manifestaron que a pesar de todo el proceso vivido, había llegado un momento en cual ya han aceptado la situación, sentían alivio después de lo vivido, lograron solucionar situaciones y agradecen algunas consecuencias positivas que dejo la pandemia, debido a que lograron aprovechar en algunos momentos aunque también se puede visualizar que no todo es bueno y llegan a manifestar desesperación y quejas. Expresando también sentimientos de añoranza.

*PA: “-es un hábito de higiene… Pero ahorita si ya ha sido más intenso ese tema. O el hecho de llegar, quitarse la ropa o sanitizar. Yo creo que eso es de que tomar conciencia de que nos debemos de cuidar y en base de ello vamos a tener algo positivo. Porque por ejemplo él y yo nos enfermábamos bastante de catarrito, tos, del estómago, nos enfermábamos seguido. Y ahorita no…” “-…ahorita es llegar y lavarse las manos y si te tienes que cambiar, cambiarte la ropa, los zapatos los dejas ahí, yo creo que en eso nos marcó bastante y el aislamiento de que disminuyó la vida social” (entrevista tres, página 6, líneas 7 a 9)*

*PV: “…conforme pasa el tiempo, han aumentado los problemas, las peleas, eh pues yo de por si sufro de depresión, entonces ya la depresión en vez de que se vaya, se mantiene, se ha mantenido más” (entrevista uno, página 1, líneas 5 a 7)*

*PC: - “ya quiero que me dé el aire”, pues obviamente aquí en México es más complicado por el tráfico como las rutinas, pero a veces sí ya me dice “no, ya extraño” porque pues él a su trabajo se hace una hora, de regreso es otra hora. Pero sí a veces si ya me dice “no, ya, ya extraño como que ver gente” hasta me dice: “ya extraño hasta lo que me caían mal” (entrevista cuatro, página 4, líneas 4 a 8)*

*PA: “…el día de mañana, aunque tengas trabajo, aunque tengas lo que tengas pues si no tienes salud pues se acaba todo. Entonces pues ahorita lo que nos está dejando la pandemia es llegar a apreciar nuestra salud como lo central para poder seguir funcionando en todo” (entrevista tres, página 6, línea 17; página 7, líneas 1 y 2)*

*PV: “digamos que la paciencia no es nuestra mejor virtud en estos tiempos. Ya como que estamos muy friccionados, a la primera de cambio empezamos a discutir. Sí ha cambiado bastante a comparación de antes. (entrevista uno, página 3, líneas 4 a 6)*

*PV: “Pues él se va a trabajar, por lo mismo del confinamiento nada más trabaja lunes, miércoles y viernes. Los otros dos días su empresa le permite pues resguardarse para evitar que se contagie, pero eso también ha dado más a que nos veamos la cara todo el día y pues más discusiones, más roces” (entrevista uno, página 4, líneas 9 a 12)*

*PA: “Yo creo que por el hecho de estar aquí juntos pues me hacía sentir tranquila, el hecho de decir: “pues no se está exponiendo, estamos aquí juntos”. Yo a él sí lo veía tristón…” (entrevista tres, página 5, líneas 1 y 2)*

## Discusión

Los hallazgos obtenidos en la investigación fueron fundamentales para analizar las relaciones y procesos que viven las familias ante el confinamiento por COVID-19, donde las participantes expresaron que durante la pandemia su familia vivió un proceso de transición para poder adquirir nuevas formas de vida, las cuales incluían hábitos y costumbres que cubrieran sus necesidades requeridas en ese momento, este proceso incluyó diferentes etapas (afrontamiento, adaptación y adopción).

El proceso de cambio por el que atraviesan las familias que viven en confinamiento por COVID-19 ha generado cambios en la rutina de los individuos, en las dinámicas familiares, en la forma de trabajar, estudiar y de vivir la cotidianidad, las familias tuvieron que reacomodar su funcionamiento, poniendo en acción medidas que les permitieran enfrentar los acontecimientos estresantes, tanto la incertidumbre frente a la posibilidad de contagio como al aislamiento estos elementos coinciden con las aportaciones de Moya y Willies, 2020^18^, y Mariela, López y Morales 2020^19^.

Durante la primera etapa, donde las familias afrontaron el inicio la pandemia, las participantes expresaron experimentar sentimientos negativos como miedo, angustia, temores y desamino, sin embargo, las participantes de este estudio también refirieron presentar sentimientos positivos como el apoyo familiar, lo que coincide con Eke, Dee, Fuchs, 2020^20^.

Respecto a la segunda etapa del proceso que vivieron las familias se encuentra la adaptación a la nueva normalidad, las familias desarrollaron estrategias que les permitieron enfrentar las situaciones estresantes, lo que a su vez genera la adopción de hábitos, costumbres y roles, estos resultados coinciden con Morales 2020^21^.

Las participantes de este estudio también expresaron actitudes positivas como responsabilidad y aprovechamiento, resultados que se asemejan a lo evidenciado por Johnson, Saletti y Tumas, 2020^22^. También se expresaron actitudes negativas como el estrés, coincidiendo con Zamarripa, Delgado, Morquecho, Cruz y Duarte, 2020^23^.

En relación a los sentimientos de frustración y negación que se mostraron durante el aislamiento provocaron un impacto emocional negativo debido a que se observa que prevalecen los sentimientos de soledad, frustración, miedo e incertidumbre, desesperación, y sobre todo la tensión emocional que provoca en ellos la pandemia, resultados similares obtuvieron Bravo y Oviedo, 2020^24^.

Finalmente, la última etapa que atraviesa la familia es la adopción de hábitos y costumbres, se apropian de los cambios adquiridos como consecuencia de la pandemia. Las madres participantes tienen manifestaciones positivas como asimilación de la situación, agradecimiento y valoración, resultados similares coinciden con el estudio realizado por Sandín, García, y Chorot 2020^25^. Muchos de los participantes han experimentado efectos como valorar nuevos aspectos de la vida, conocer o vivir nuevas experiencias positivas, aprender a valorar cosas importantes de las que antes no era consciente, o descubrir nuevas capacidades o aficiones.

El cómo se está viviendo la contingencia sanitaria por COVID-19 es distinto a las diferentes pandemias a lo largo de la historia, como la de H1N1, de acuerdo a Estrada, 2009^26^ el confinamiento fue más corto y las prioridades familiares fueron distintas. El proceso que han mostrado las familias mexicanas ante el COVID-19 en España se vive diferente como menciona Lacomba, et. al., 2021^10^ ya que al inicio España fue uno de los países más afectados, la convivencia en los hogares fue de las afectadas ya que se volvió estresante debido a la falta de libertad con la que viven día a día, sin embrago coinciden en otros aspectos como, la perdida de trabajo, el teletrabajo, el tener que realizar las labores del hogar, la supervisión de las tareas escolares, el cuidado de sus hijos y trabajar al mismo tiempo.

En Cuba, su sistema de salud es muy diferente, y eso ha contribuido a que la manera en que se vive sea distinta, de acuerdo a lo que refiere Ribot, Chang y González en 2020^27^ desde el inicio de la pandemia se implementó un protocolo de actuación, con alcance nacional, con vista a la prevención, control y manejo del caos, donde se incluyen el impacto en la rutina socio-familiar; González y Hernández, 2021^28^ hacen referencia a en países asiáticos debido a su que tienen diferentes sistemas, se pudo otorgar ayudas económicas directas a los ciudadanos y a las familias, en Corea del Sur aprobó el pago en efectivo de emergencia a todas las familias excepto aquellas con mayores posibilidades económicas, lo que disminuyo el estrés en las familias.

El cómo viven las familias el confinamiento también se ve afectado por las desigualdades económicas, como por ejemplo en el cierre de escuelas, de acuerdo a Cifuentes, 2020^6^; los integrantes de las familias no cuentan con los espacios suficientes para desarrollar sus actividades escolares y/o extraescolares.

### Limitaciones

El estudio presento limitaciones debido a la muestra y el número de entrevistas realizadas, por lo tanto, no se pueden universalizar los resultados a otras poblaciones, ni la presunción de saturación de datos. En relación a las entrevistas, no se realizaron de forma personal, pudiendo limitar la observación de las expresiones, movimientos y gestos de las participantes. Los participantes se concentraron en áreas urbanas y por las características de las familias acorde a lo descrito por Duvall en 1977^29^, el estudio únicamente tomo en cuenta dos etapas del ciclo vital familiar el de familia con hijos preescolares y escolares.

## Conclusiones

Las participantes expresaron que durante la pandemia por COVID-19, la familia experimentó un proceso de transición para poder adquirir nuevas formas de vida, las cuales incluían hábitos y costumbres que cubrían las necesidades requeridas en ese momento, en este proceso se pudieron identificar diferentes etapas (afrontamiento, adaptación y adopción). Las familias afrontaron la situación mediante sentimientos positivos como el apoyo entre los integrantes de la familia y la asimilación de los cambios que se experimentaron, sin embargo, estos cambios también generaron actitudes negativas como estrés y frustración.

En la segunda etapa identificada las familias se adaptaron a nuevos comportamientos, roles y rutinas para poder buscar las mejores estrategias y enfrentar situaciones complicadas. Finalmente, la última etapa identificada fue la adopción, en esta etapa la familia ha adquirió hábitos y costumbres que resolvieron las necesidades de la vida cotidiana para poder cumplir con la nueva normalidad.

Bajo este análisis, se ha permitido conocer el importante papel de la familia en tiempos de pandemia y el proceso por el que atravesaron para poder encontrar un equilibrio para la situación. Eventualmente habrá que cuestionarse si el proceso aquí descrito se vive en familias que se encuentran en otras etapas del ciclo vital familiar y ante situaciones de salud diversas como en estados crónicos o agudos.
